# Oxytocin and Major Depressive Disorder: Experimental and Clinical Evidence for Links to Aetiology and Possible Treatment

**DOI:** 10.3390/ph3030702

**Published:** 2010-03-16

**Authors:** David A. Slattery, Inga D. Neumann

**Affiliations:** Department of Behavioural and Molecular Neuroendocrinology, University of Regensburg, Universitätsstr 31, Regensburg D-93053, Germany

**Keywords:** neuropeptide, depression, oxytocin, social attachment, early-life

## Abstract

Affective disorders represent the most common psychiatric diseases, with substantial co-morbidity existing between major depressive disorders (MDD) and anxiety disorders. The lack of truly novel acting compounds has led to non-monoaminergic based research and hypotheses in recent years. The large number of brain neuropeptides, characterized by discrete synthesis sites and multiple receptors, represent likely research candidates for novel therapeutic targets. The present review summarises the available preclinical and human evidence regarding the neuropeptide, oxytocin, and its implications in the aetiology and treatment of MDD. While the evidence is not conclusive at present additional studies are warranted to determine whether OXT may be of therapeutic benefit in subsets of MDD patients such as those with comorbid anxiety symptoms and low levels of social attachment.

## 1. Introduction

Major depressive disorder (MDD) is a major health concern, with lifetime prevalence in the United States estimated to be as high as 16.2% [1]. Furthermore, the World Health Organisation states that MDD is the second largest global health burden [2,3]. Despite a biological basis for MDD being postulated as early as the 5^th^ century BC by Hippocrates, together with substantial research efforts, the underlying aetiology of mood disorders remains poorly elucidated [4,5,6]. Currently, according to the *Diagnostic and Statistical Manual of Mental Disorders Edition IV*, only depressed mood and anhedonia (loss of interest or pleasure in previously rewarding stimuli) are considered as core symptoms of MDD. However, there are also prominent additional symptoms, at least in subsets of depressed patients, which include sleep disturbance [7], anxiety and sexual dysfunction [8]. 

Although a number of pharmacological agents are available to treat depression, approximately 30–40% of patients do not respond to these [9] and in those who do benefit there is a delayed onset of action. Therefore, a major emphasis in modern psychiatric research is to uncover the underlying aetiology of mood disorders, and to develop novel efficacious antidepressant treatments. Neuropeptides, which have discrete synthesis and receptor sites, have emerged as viable research candidates, with respect to both the pathophysiology and treatment of MDD. In this context, interest has mainly focussed on the brain neuropeptides corticotrophin releasing hormone (CRH) and vasopressin (AVP), which both exert anxiogenic and depression-like effects. Numerous review papers are recommended for the interested reader [10,11,12,13]. However, the nonapeptide oxytocin (OXT), which only differs from AVP by two amino acids, has recently received a great deal of interest in complex behaviours and is the focus of this review.

Originally considered as a “maternal hormone” based on its role in the regulation of reproductive functions and maternal behaviour, OXT has recently been implicated in a plethora of other behaviours and neurochemical processes (see [14] for a review). OXT is mainly synthesised in magnocellular neurons of the hypothalamic paraventricular (PVN) and supraoptic (SON) nuclei and is secreted into the periphery *via* the posterior pituitary where it mediates effects on uterine contractions, milk ejection in lactation and cardiovascular control, among others. These actions are mediated *via* the OXT receptor (OXT-R) of which only one has been identified to date. The receptor is a member of the G protein-coupled receptor (GPCR) family and is coupled to phospholipase C [15]. In addition, there is widespread distribution of the OXT-R throughout the central nervous system and independent central and peripheral release of OXT has been shown [16]. Recent evidence has implicated OXT in numerous complex behaviours, particularly anxiolysis [17,18,19,20,21]. Thus, given that half of the patients diagnosed with MDD meet criteria for co-morbid anxiety disorder [8], OXT may be of therapeutic benefit in these patients, as well as those with only anxiety. The following review will assess the literature on other potential roles of OXT in the pathophysiology and treatment of MDD.

## 2. Evidence from Animal Studies

### 2.1. Animal models of depression

The first report of an antidepressant-like effect of OXT came from Arletti and Bertolini [22] who demonstrated that intra-peritoneal (i.p.) administration both, acutely, and after ten daily-repeated injections decreased immobility time in the forced swim test (FST) in mice: the most widely utilised animal model of depression [4]. The FST results were recapitulated in aged (26 month) Wistar rats following i.p OXT administration [23]. In addition, subcutaneous OXT was reported to decrease the number of escape failures in the learned helpless test [24], which is indicative of an antidepressant-like effect [25]. In these studies OXT was given peripherally, but we have to keep in mind that neuropeptides like OXT can only cross the blood-brain barrier (BBB) in very low amounts when supra-physiological concentrations of exogenous OXT are administered in adult rodents [26,27]. However, it is also possible that active OXT fragments can cross the BBB to produce central effects [28]. In support, findings of other OXT-mediated effects, such as decreased blood pressure can also be observed following both peripheral and central OXT administration [29]. Recently, the FST findings were recapitulated in mice in the tail suspension test (TST), a test with a similar construct [30]. Furthermore, the antidepressant-like effect was also shown after intracerebral OXT administration in the TST [30]. However, a novel non-peptidergic OXT agonist, WAY-267464, while having an anxiolytic profile, did not replicate the OXT effect in the FST [30]. These results suggest that, at least in mice, systemic or central administration of OXT has antidepressant-like properties. 

In rats exposure to forced swimming stimulates the release of OXT within the central amygdala (CeA) again suggesting a link between OXT and depression. However, local administration of an OXT-R antagonist (OXT-A) revealed that the OXT release was linked to a more passive stress-coping style, *i.e.,* depressive-like behaviour [31]. As central OXT actions have also been linked to the promotion of sleep [32] in male rats, the possibility exists that brain OXT contributes to a generally reduced arousal and activity. While this supports a pro-depressive effect of OXT, this may be region specific as we recently demonstrated in animals selectively bred for high or low anxiety-related behaviour [33] that neither acute nor chronic icv OXT infusion altered behaviour in the FST [20]. Taken together, the present evidence from the few studies assessing OXT in animal models of depression is inconclusive regarding a potential antidepressant-like effect of OXT. More studies are needed in order to determine whether the findings in the early studies can be replicated and extended to central administration and additional models. These previous studies have only examined the effect of OXT in the FST and TST, or after peripheral administration. Therefore, studies assessing the potential role of central OXT in models that assess different endophenotypes are warranted. For example, replication of the learned helplessness results following central administration, anhedonia (see below), or in sleep disturbances, and in such tests following chronic stress exposure would provide better indication of any potential antidepressant-like properties of OXT.

### 2.2. OXT and the reward system

As mentioned above anhedonia is a core symptom of depression and it is also one, which is easily assessable in animal studies [4]. The conditioned place preference paradigm is widely used to study drugs of abuse, such as cocaine. Here, animals spend preferentially more time in an environment that has previously been associated with the drug indicative of its rewarding properties. OXT, when infused subcutaneously in rats, was shown to result in a place preference, suggesting that peripheral infusion of OXT results in a positive hedonic state [34]. Contrastingly, a more recent study assessing intracerebral OXT infusion did not replicate these findings [35]; although the latter study was performed in mice. However, OXT injection into the ventral tegmental area (VTA), ventral subiculum and posteromedial cortical nucleus of the amygdala causes increased dopamine release in the nucleus accumbens [36,37]. This again suggests that OXT may itself have rewarding properties given that drugs of abuse also result in increased DA release in this region [38].

The brain OXT system shows high activation during the peripartum period with increased synthesis, intracerebral release in response to birth and suckling and receptor presence, and OXT is known to be an important mediator of maternal behaviour [39,40]. In a recent fMRI study, suckling was shown to increase activity in numerous regions of the reward circuitry in lactating rat dams; particularly the VTA-accumbens-prefrontal cortical pathway [41]. This increase was prevented by pre-administration of an OXT-A, and suggests that suckling is rewarding for the mother *via* OXT release, encouraging her to continue engaging in the behaviour. This finding also fits with a series of experiments from Morrell and co-workers showing that, in early lactation, dams find pups more rewarding than cocaine but that this reverses in mid-to-late lactation [42,43]. These findings suggest that OXT, either via exogenous application or endogenous release in response to specific stimuli, results in a positive hedonic state. Therefore, OXT may help to alleviate the severity of anhedonia observed in MDD patients. Moreover, they suggest that altered OXT system activity may be involved in postpartum depression (see OXT in the peripartum period section).

There is also high co-morbidity between MDD and drug abuse, and withdrawal from such drugs leads to the appearance of many symptoms observed in MDD. This has led some authors to postulate that similar neurobiological mechanisms may underlie the disorders [44]. Low doses of 3,4-methylene-dioxymethamphetamine (MDMA; ecstasy) have been shown to increase the level of plasma OXT in both rodents and humans, and administration of an OXT-A was shown to prevent the increased social behaviour induced by MDMA in rodents (reviewed in [45]). This suggests that OXT may also be involved in the rewarding effects of acute MDMA, again supporting a role of OXT in reward and positive hedonic states. Contrastingly, the literature suggests that OXT actually decreases abuse and withdrawal symptoms of drugs such as cocaine and methamphetamine [35,46]. For example, icv administration of OXT decreased the abuse of opiates and cocaine in rodents [47,48]. OXT has also been shown to decrease methamphetamine-induced hyperactivity and place preference by altering mesolimbic DA turnover [35,49]. Similar findings have been reported regarding OXT preventing the increase in DA release following apomorphine [50], cocaine [47] and morphine [46] administration. Importantly, OXT was also able to prevent the reinstatement of methamphetamine-place preference induced by restraint stress but not by methamphetamine administration [35]. These findings suggest that OXT works to prevent the reinstatement of drugs by a stressor, which is in agreement with the known hypothalamic-pituitary-adrenal (HPA) axis attenuating properties of OXT (see below). A potential reason for the ability of OXT infusion to decrease the abuse potential of drugs of abuse and withdrawal symptoms is the reported decrease in OXT mRNA and OXT-fibers in the nucleus accumbens and VTA following seven days of Δ-9-tetrahydrocannabinol administration [51] or in the PVN and SON following morphine infusion [52]. This contrasts to the increased excitability of OXT neurones in the SON precipitated by naloxone infusion in morphine-withdrawal in rats [53]. However, these findings again implicate alterations in the OXT system underlying physiological changes as a result of drug withdrawal. Additionally, lithium administration was shown to prevent the withdrawal symptoms from cannabinoids by increasing OXT mRNA expression in the PVN and SON [54]. When an OXT-A was given, lithium administration was no longer able to prevent the withdrawal syndrome, while icv OXT was able to mimic the lithium effects alone [54]. Therefore, OXT could represent a viable treatment option in patients with co-morbid drug abuse, and also to reduce the depressive-like symptoms precipitated by drug withdrawal, which can in turn lead to the development of MDD [44]. Studies using intra-cranial self stimulation under basal and drug-withdrawal conditions could provide a greater insight into the postulated role of OXT in anhedonia [55]. Similarly, studies assessing central OXT on sucrose preference after chronic stress with an antidepressant as a control could determine whether central OXT could attenuate the anhedonic-phenotype observed in such paradigms.

### 2.3. OXT and social interaction

Social interactions and support have been shown to result in positive health benefits, while a lack, or perceived lack, of social support is associated with increased likelihood of MDD and cardiovascular diseases. There is a great deal of evidence in the literature demonstrating a role of OXT in many aspects of social behaviour, including pair bonding, sexual behaviour and mother-offspring interactions (see [14,56,57,58,59] for extensive reviews). Perhaps the best known example is the difference in OXT-R expression between monogamous and non-monogamous female voles. Monogamous prairie voles were shown to have higher densities of OXT-R in the caudate putamen and nucleus accumbens compared with non-monogamous montane voles. Infusion of an OXT-A into the prefrontal cortex and nucleus accumbens, but only in females, decreased mating-induced partner preference [59]. OXT knockout mice also have altered social interactions, failing to recognise a familiar con-specific, which can be reversed by intra-medial amygdala infusion of OXT [60]. These findings suggest that OXT may be of importance for both the development of social withdrawal/anxiety in MDD and that exogenous OXT may be of therapeutic benefit in MDD patients with low attachment security. In support, OXT has been shown to be altered in the plasma and PVN after four weeks of social isolation in highly social female prairie voles [61]. Furthermore, social isolation was shown to result in reduced sucrose intake and preference, indicative of anhedonia, and increased depression-related behaviour in the FST [62]. These alterations were prevented by long-term peripheral OXT administration (during the third and fourth week of isolation), as were a number of cardiac parameters that were also shown to be altered [62]. In all of these tests, OXT administration had no effect in animals paired with a sibling suggesting that it specifically reversed isolation-induced behavioural and autonomic deficits. Therefore, it is possible that OXT administration would be of therapeutic benefit to reverse the decreased sociality observed in MDD patients. In turn, this could result in the benefits known to occur from positive social interactions. Moreover, decreased OXT system activity may lead to the development of social withdrawal in MDD and also increase the risk of psychiatric disorders (see also OXT and social interactions in clinical section). Thus, it would be of interest to assess the role of OXT in models of social defeat, which result in reduced social interaction, believed to be reflective of a depressed phenotype, to examine this possibility [63].

### 2.4. OXT and 5-HT interactions

The monoamine hypothesis of depression predominated much scientific research into MDD, both academic and pharmaceutical, from the 1960s until recently [6]. Although new hypotheses have been postulated, all currently approved antidepressants with the exception of agomelatin, the melatonin-based compound that was licensed last year, have a primary mechanism of action of increasing the activity of monoamine systems. Therefore, a possible mechanism for a role of OXT in the pathophysiology and treatment of MDD is its interaction with the serotonergic system. It was shown in conscious rat dams that 5-HT injections potentiated the regular OXT bursts that occur in response to the suckling stimulus as assessed by electrical activity of OXTergic neurones [64]. Moreover, 5-HT receptor antagonist treatment or 5-HT depletion by administration of *p*-chlorophenylalanine (pCPA) inhibited the suckling response. D-Fenfluramine, a 5-HT releasing agent, has also been reported to increase c-fos expression in OXT neurones of both the PVN and SON [65]. These effects may be mediated by 5-HT1A- and 2A receptors as specific agonists for these receptors were shown to dose-dependently increased plasma OXT levels. The effects of the agonists were blocked by pre-treatment with NAN-190 (a 5-HT1A receptor antagonist) or ritanserin (5-HT2A/2C) compounds, respectively [66,67]. Interestingly, plasma AVP levels were not altered by activation of either receptor subtype, supporting a specific role of the serotonergic system for manipulating OXT release [66,67]. The stimulatory effect of activation of the serotonergic system appears to occur also at the level of gene transcription as 5-HT1A- 1B, - 2A- and 2C receptor agonist administration was shown to increase OXT mRNA expression in the PVN and SON [68]. These data are also in agreement with evidence showing that stimulation of the dorsal raphe nucleus leads to an increase in neuronal activity of magnocellular PVN neurones [69]. In a series of lesion studies, *p*-chloroamphetamine and fenfluramine, increased plasma OXT levels in control and SON-lesioned animals, but not in those with PVN-lesions. Interestingly, the effects in control animals were prevented by administration of fluoxetine, which suggests that, at least acutely, re-uptake of 5-HT into the nerve terminals is required to stimulate OXT secretion into the plasma [70]. In contrast to these findings a study assessing the effect of i.p. citalopram, a more selective 5-HTT blocker than fluoxetine, and a structurally different SSRI, zimeldine, on plasma OXT levels revealed an increase following both acute and chronic (2 week) administration [71]. Further anatomical evidence was shown by Emiliano *et al*., [72] who demonstrated that the density of 5-HTT fibres followed the distribution of OXT-containing neurones in the PVN and SON in macaques. Together with some of the data outlined above, this observation lead the authors to speculate that efficacy of SSRI’s to restore social interactions/bonding may be in part due to the activation of the OXT system [72]. 

One of the main reasons in the clinical setting for patients stopping SSRI treatment is the loss of libido and anorgasmia caused by their use. Sexual stimulation in men and women causes an increase in plasma OXT levels [73,74]. In male rats, successful mating is accompanied by increased intra-PVN-release of OXT, and such central OXT is necessary for male sexual behaviour [75] and the anxiolytic effect of mating [76]. Chronic fluoxetine administration in male rats has also been shown to decrease ejaculation frequency and administration of OXT prior to the test specifically prevented this effect [77]. This data fit with that of Li *et al*., [78,79,80] showing that chronic fluoxetine administration decreased the affinity of 5-HT1A- and 2A-receptors on OXT neurones. Therefore, it is possible that an initial increase in OXT system activity by SSRI treatment gives way to a decrease following chronic up-regulation of the serotonergic system. Thus combining SSRI treatment with OXT may help to decrease the coincidence of sexual dysfunction caused by the former and, therefore, greater tolerability of the patients to continue with the antidepressant treatment. 

There has also been recent evidence showing that the well-documented anxiolytic action of OXT may be mediated via OXT-induced 5-HT receptor activation [81]. In this elegant study, a yellow fluorescent protein, *Venus*, was placed under control of the regulatory region of the gene encoding the OXT-R in order to determine the expression pattern of OXT-R throughout the brain [81]. In these mice, widespread expression of OXT-R-containing cells was observed but most strikingly a large co-localisation of *Venus* with tryptophan hydroxylase-positive neurones in the raphe nuclei was shown [81]. Intra-raphe infusion of OXT increased 5-HT release within the median raphe, which was inhibited by 5-HT2A/2C receptor antagonists. This led the authors to speculate that the increased serotonergic activity induced by OXT may underlie its anxiolytic effects [81], and given the use of SSRI’s in MDD, these findings may also relate to their antidepressant-like effects. It would be interesting to determine whether the increased 5-HT release is maintaining following chronic OXT administration, and therefore, support another possible role for OXT in the treatment of MDD.

### 2.5. OXT and stress responsivity

Numerous stressors including both social and physical, have been shown to increase central OXT release in regions known to be involved in stress-responsivity including the PVN, CeA and lateral septum [31,82,83,84,85,86]. Such local release has been linked to an inhibitory effect of OXT on HPA axis (re)activity in male and virgin female rats [18,87]. We could show that OXT neurons respond to emotional, physical, or pharmacological stressors with elevated peripheral and somato-dendritic release within the hypothalamus and the amygdala in male and virgin female rats [14]. Endogenous OXT has also been shown to inhibit the activity of the HPA axis locally in the PVN, amygdala, or medio-lateral septum [18]. In support, in mice lacking OXT, exposure to stress has been shown to lead to an increase in CRH mRNA expression within the PVN [88] and elevated corticosterone levels [89]; indicative of an elevated stress response [90]. The time around birth is accompanied by complex behavioural, physiological and neuronal adaptations of the maternal brain, which ensure reproductive functions and the mental health of the mother. Two major adaptations are the attenuation of the HPA axis to a variety of stressors and anxiolysis, which are mediated, at least in part, via the increased activity of the OXT system [40]. This suggests that OXT may mediate stress-hyporesponsiveness over a longer duration than those observed in the acute studies mentioned above. Additional support comes from a study showing that chronic icv OXT administration (10 ng/h; 7 day) decreases neuronal and neuroendocrine responses to acute restraint stress exposure in ovariectomized virgin rats [91]. Chronic hypertonic saline has been shown to increase OXT in the PVN [92] but surprisingly, there have been very few other studies examining the effect of chronic psychological stress on the OXT system [93,94] with the exception of the aforementioned studies on effects of isolation stress in female prairie voles [60,62]. Taken together, these findings suggest that the OXT system acts as a stress-buffering factor, at least to acute stimuli. Therefore, given that, long-term and especially chronic stress is a well-documented risk factor for the development of psychiatric disorders in predisposed individuals; contribution of a dysfunctional OT system is suggested (see below). Additionally, these studies suggest that OXT may represent a therapeutic benefit in MDD patients with abnormal stress responses, such as observed in patients with paradoxical outcome of the dexamethasone suppression/CRH challenge test [95]. However, it is clear that more studies are required to assess the role of chronic stress on the OXT system and whether chronic OXT administration would be beneficial in protecting against the detrimental effects of chronic stress exposure.

## 3. Evidence from Human Studies

### 3.1. OXT in the peripartum period

Similar to the findings from animal studies, OXT has been implicated in decreasing stress-responsivity in humans. Indeed, a series of experiments performed in the 1980s revealed that systemic OXT administration attenuated both basal and pharmacologically-induced ACTH and cortisol responses in healthy volunteers (summarised in [96]). Breast-feeding was shown to increase plasma OXT in women [97,98], likely to be accompanied by central OXT release [39,99]. Accordingly, Heinrichs *et al*., [96] determined whether breastfeeding prior to an acute stressor exposure may underlie the decreased stress responsivity observed in the peripartum period. Lactating women were asked to either breast-feed or hold their child for a 15 min period, 30 min before they were subjected to the Trier Social Stress Test (TSST) in which they have to give an unprepared speech and perform mental arithmetic in front of an audience [96]. Breast-feeding attenuated the TSST-induced plasma cortisol rise when compared with the lactating holding group [96] indicating a possible involvement of an activated brain OXT system [39] along with many other alterations. Additionally, a recent study in first-time mothers showed that those who had low adult attachment ratings (a risk factor for MDD) prior to birth also displayed lower activation of the reward circuitry and lower plasma OXT levels when interacting with their infants [100]. These mothers also displayed the lowest attachment to their infants. This suggests that a certain level of activity of the OXT system, which, in humans, can only be estimated by plasma (or CSF) concentrations, is required for the rewarding aspects of adult attachment and mother-infant relations. Plasma OXT levels are also decreased in cocaine-addicted mothers who are more likely to have psychiatric disorders and less attachment to their infants [101]. Together these studies suggest that OXT is important for the decreased stress reactivity observed in lactation and general reward-related behaviour, and as such represent an important factor in the aetiology of postpartum depression (PPD). To date, the underlying mechanisms of PPD remain poorly elucidated but it has been suggested that altered activity of the OXT system may play an important role [40]. Moreover, given the importance of early-life experiences on adult mental health these findings also suggest that infants of mothers with low OXT system activity may be more vulnerable to develop MDD or related disorders in adulthood (see next section). Future clinical studies could examine whether OXT could be of therapeutic value in PPD, and longer term to less the risk of the development of psychiatric disorders in the children of such mothers.

### 3.2. OXT and adverse early-life events

Early life stress has repeatedly been demonstrated in preclinical findings to result in increase anxiety- and/or depression-related behaviour and the severity of the response to stress exposure in adulthood [102,103]. Moreover, in rodent studies, stress to the dam resulted in increased anxiety-related behaviour in the offspring and lower levels of OXT-R compared with pups from similar high-caring mothers [104]. These findings suggest that negative early-life events may lead to alterations in the OXT system, which mediate, at least in part, the increased risk of developing psychiatric disorders in adulthood. 

Similarly, numerous human studies have implicated negative early-life experiences to be a risk factor for the development of psychiatric disorders in adulthood (see [105] for a review). In contrast, positive social interactions have been shown to promote health and protect against the development of psychiatric disorders [106,107]. It has also been shown that adults who suffer from a lack of social contact with their parents in childhood, such as may occur following a divorce or a death, are also at an increased risk for the development of psychiatric disorders [108]. A pilot study revealed that men who had experienced disrupted early-life parenting demonstrated less sensitivity to the basal plasma cortisol-reducing effect of intra-nasal OXT [109]. Intra-nasal administration of neuropeptides has previously been shown to cause a resultant increase in their CSF levels [110]. Therefore, it is possible that adverse early-life experiences resulted in altered activity of the brain OXT system in adulthood, especially its receptors, and thereby, increase the likelihood of developing mental disorders. In support, OXT has recently been shown to increase the feeling of attachment security in adult males who suffer from insecure attachment patterns, which are usually the result of early-life experiences and can also lead to the development of MDD [111,112]. Furthermore, a positive correlation has recently been reported in a group of MDD patients with high levels of separation anxiety and a single-nucleotide polymorphism in the OXT-R [113]. In a recent study, 22 females with no psychiatric illness who had experienced childhood traumas were shown to have lower levels of CSF OXT compared with controls [114]. Although not suffering from psychiatric disorder, there was also a negative correlation observed between CSF OXT levels and current anxiety ratings. These findings suggest that the decreased OXT activity may be a susceptibility factor for the increased likelihood of developing psychiatric disorders including MDD following adverse early-life experiences. 　

### 3.3. OXT and social interactions

In support of the findings reported in the previous section, OXT has been implicated in a series of studies to play an important role in mediating the positive effects of social support on buffering the response to stress [107]. The extent of HPA axis and fear responses to the TSST was shown to be reduced by social support prior to the test [115]. Given the extensive animal literature, Heinrichs *et al*., [116] assessed whether OXT may, at least in part, mediate this response. Therefore, healthy men were given social support, *i.e.,* the company of their best friend (or no support) and intra-nasal OXT (or vehicle) before being subjected to the TSST. The greatest dampening effect on cortisol and anxiety-ratings occurred when social support and OXT were given in combination [116]. In continuation of these findings, a more recent study examined the effect of intra-nasal OXT on communication and cortisol levels during partner conflict [117]. Intra-nasal OXT, in both men and women, increased the duration of positive behaviour such as eye contact and emotional self-disclosure compared with negative behaviour (e.g., contempt or defensiveness) suggesting a lack of sexual dimorphism in this effect. In addition, OXT attenuated the increase in salivary cortisol levels induced by the conflict, which suggests that it not only improves positive social interaction, but also diminishes the physiological reactivity to social stressors [117]. The ability of OXT to decrease social stress reactivity suggests that altered activity of the OXT system may be involved in the development of MDD triggered by social stressors in vulnerable individuals. Additionally, they suggest that OXT administration in MDD may promote positive social interaction and, thus, may consequently lead to symptom improvement.

A series of human studies have shown that intra-nasal administration of OXT has improves processing of facial cues, both positive and negative, as well as decreasing amygdala activity to fearful faces [118,119,120,121]. These findings are in agreement with studies assessing the SSRIs, citalopram and reboxetine, in similar paradigms [122,123,124]. Therefore, OXT treatment may reduce the salience of potentially threatening social cues possibly via interactions with the serotonergic system. These findings suggest, together with the other data relating to OXT improving social interactions that OXT may be useful as an adjunctive agent, in MDD patients, especially in those with high ratings in social components and social phobia. However, while the reported behavioral effects of intranasal OXT have been consistent, none of these studies have measured CSF levels of this peptide after intranasal administration. Therefore, future studies should examine whether intranasal OXT improves such social behaviours with a corresponding increase in intra-cerebral (or CSF) levels of OXT.

### 3.4. MDD and plasma OXT

A number of clinical studies have attempted to correlate the level of circulating plasma OXT with depressive symptomology. In the first such studies reduced plasma OXT concentrations were observed in MDD patients compared with controls [125,126]. A similar finding was recently reported for a female cohort of depressed patients but was not confirmed in males [127]. However, in the latter study, the number of patients in the male cohort was very low compared with women and may have lead to the negative finding. The patients were then treated with electroconvulsive therapy (ECT), venlafaxine, an SSRI, or a tricyclic antidepressant and after a positive response as measured by the Hamilton Depression Rating Scale (HDRS), plasma OXT levels were re-assessed. However, no alterations in plasma OXT levels were observed. This again may be due to the low number of patients as well as the treatments, which were not separately analysed but grouped together. It would be interesting to perform a similar study with a larger cohort of patients given the earlier findings and also the animal literature suggesting a link between OXT and SSRIs. A study by Van Londen *et al*., [128] comparing 52 MDD patients with 37 controls found no difference in mean plasma OXT levels between the groups but did observe more variation within the MDD group. Similarly, Cyranowski *et al*., [129] reported greater variability in pulsatile OXT release over two 1 h sessions in women with MDD compared with controls. This may suggest an altered circadian release of OXT in MDD but no follow-up studies have examined this hypothesis. In addition, OXT concentrations obtained during an affiliation-associated imagery session positively correlated with the severity of MDD in this group again suggesting an association between the OXT system and sociality in depressed patients [129]. Further, in a group of 60 depressed outpatients of mixed heterogeneity, plasma OXT levels were assessed in relation to a Temperament and Character inventory, which assesses reward dependence, novelty seeking, harm avoidance and persistence. In this sample set a positive correlation between plasma OXT and reward dependency and novelty seeking was found [130]. A negative correlation between OXT and depression- and anxiety- symptom severity in depressed patients has also been reported [131]. Therefore, it appears that plasma OXT may be correlated with a number of symptom clusters in MDD. Additionally, and particularly in women, plasma OXT levels may be lower or more varied in MDD compared with controls but larger studies assessing plasma OXT across the whole day are required to assess this. While further studies on the role of OXT and AVP in human social behaviour are warranted, it is not obvious that measurement of peptides in the periphery will provide any useful information.

### 3.5. MDD and CSF OXT

For the interpretation of human plasma OXT concentrations we have to keep in mind that intracerebal and peripheral release of neurohypophysial neuropeptides like OXT and AVP may occur in a co-ordinated or independent manner depending on the quality of the stimulus (for review see [85]. Consequently, alterations in plasma OXT levels do not necessarily reflect similar alterations in the (locally specific) activity of the brain OXT system. However, in strict contrast to the dynamics of central versus peripheral AVP release, to date, most physiological, stressful or pharmacological stimuli, which were shown to trigger central OXT release were also identified to induce OXT secretion into the blood [84,85]. Quantification of neuropeptide content in the cerebrospinal fluid (CSF) integrates processes of central release, diffusion and degradation of OXT, and should, therefore, be a better assessment of the activity of a given brain neuropeptide system.

A number of studies have, therefore, assessed the OXT concentration in the CSF of depressed patients. Demitrack and Gold [132] found no significant difference in CSF levels of OXT between MDD and control subjects.Pitts *et al*., [133] assessed CSF levels of OXT in a heterogeneous population of MDD patients (10 men and nine woman) who were further separated into dexamethasone non-suppressors and suppressors and described a trend towards lower OXT levels in the CSF of dexamethasone suppressors compared with healthy controls. Together with the studies assessing plasma OXT, these findings suggest that the OXT system may be dys-regulated in psychiatric disorders. However, larger studies are required to strengthen the association.

### 3.6. Post-mortem studies

In contrast to the majority of plasma and CSF data obtained, post-mortem studies assessing OXT neuronal number or mRNA levels have suggested that the OXT system within the hypothalamus is increased in MDD. Purba *et al*., [134] were the first to assess the number of OXT-containing neurones in the PVN of MDD and bipolar tissue samples. There was a significant increase in OXT-immunoreactivity in patients with MDD and bipolar compared with controls; while there was no difference between MDD and bipolar patients. More recently, using *in situ* hybridisation increased OXT mRNA levels where reported in the PVN, but not SON, of melancholic MDD patients compared with non-melancholic subjects [135]. While a trend was also reported for a similar increase in melancholic patients compared with controls this did not reach significance, but the small sample size may have occluded a significant finding. Following this study, Wang *et al*., [136] performed laser-capture microscopy on snap-frozen tissue to obtain PVN and SON tissue punches from seven MDD/bipolar patients and seven age-matched controls and then assessed a number of genes including OXT by quantitative PCR. Similar to the study by Meynen *et al*., [135] they found a trend towards an increase in OXT mRNA levels in the PVN of the mood disorder group compared with controls [136]. Taken together, the available data suggests that the OXT system in MDD may be over-active at the level of the PVN. However, more careful selection criteria and larger samples may help to elucidate the proposed correlations. These findings, together with those observed in plasma and CSF studies suggest that an up-regulation of OXT synthesis at the hypothalamic level may occur to compensate for the reduced OXT release. Therefore, exogenous application of OXT in MDD could restore the system and as a result have antidepressant effects. It would also be beneficial to have tools to assess the OXT system in patients with depression, such as PET or SPECT tracers. Such tools would enable the role of OXT in MDD and following antidepressant treatment to be assessed in a more timely and accurate manner.

### 3.7. OXT and Electroconvulsive therapy (ECT)

OXT also received substantial interest in relation to ECT at the start of the 1990’s. While ECT is one of the most effective treatments for severe MDD with a relatively rapid onset of action the underlying mechanism of its action is poorly understood. It was first postulated by Scott *et al*., [137] that the release of OXT in response to ECT may be indicative of a positive clinical outcome, which was supported by later studies [138,139]; although the clinical outcome was not reported in these studies. Scott and co-workers followed up their initial study by assessing the effect of ECT on plasma vasopressin-associated neurophysin I (hNpI) and OXT-associated neurophysin II (hNpII) over the course of repeated sessions. While hNpI or II release did not alter during the improvement of depressive symptoms from the first to last ECT session, hNpII secretion during the first ECT treatment correlated positively with clinical improvement [140]. However, another study assessing the relationship between ECT, plasma OXT and clinical outcome did not find any correlation between plasma OXT and clinical outcome [141]. A larger study by Devanand *et al*., [142] employing 55 patients observed no correlation between plasma OXT levels and the therapeutic effects of ECT. This suggests that the acute increase in plasma OXT is merely a consequence of the stressful ECT therapy experience and is not in any way related to its therapeutic benefit. In this context, estimation of the effects of ECT on central OXT via CSF levels seems to be essential in order to draw further conclusions. 

### 3.8. OXT and drug discovery

There are currently a number of clinical studies assessing the role of OXT in a number of psychiatric disorders (summarised in Table 1; further information can be found at www.clinicaltrials.gov). Although some of the current endeavours are not directly related to MDD, with further research they may become relevant to the disorder. It is of particular interest to note that many studies are assessing the role of OXT in social functions or anxiety-related disorders and if the results are positive it could lead to the study of OXT in MDD patients. These studies will provide a greater understanding of OXT in complex psychiatric disorders and hopefully, verify the information outlined in this review gathered from the preclinical and human studies performed to date. However, as stated above, such studies should also assess the level of the neuropeptide in the central compartment to validate the findings. 

**Table 1:** Current drug discovery status of OXT and endophenotypes of depression.

Study Center	Study aims
University of North Carolina	Social functioning / decrease paranoia and psychotic symptoms in schizophrenia
University of California / Stanley Medical Research Institute	Augmentation of antipsychotics in schizophrenia patients
Lawson Health Research Institute / Alzheimer’s Society London & Middlesex	Social cognition in frontotemporal dementia
Sheba Medical Center	Prevention of post-traumatic stress disorder
University of Maryland / NIMH	Social affliation, anxiety and cognition
Harvard School of Public Health / Brigham and Women’s Hospital	Amelioration of the neuroendocrine and cardiovascular effects of stress
University of California	Add-on for stable anxiety patients
NIDA / University of Maryland	Drug dependence
NIMH	Functioning of neurocognitive symptoms in mood disorders (together with AVP)
NIMH	Affectionate writing in response to acute stress

## 4. Conclusions

Overall, the evidence from animal and clinical studies suggests a potential role of the OXT system in the pathophysiology of MDD, as well as a potential therapeutic benefit of OXT in at least some subsets of MDD patients. Its significant involvement in multiple aspects of behavioural and neuroendocrine regulation including anxiolytic and anti-depressive properties, promotion of various social interactions and reward as well as attenuation of behavioural and hormonal stress responses, makes the brain OXT system an ideal candidate central to possible therapeutics. The potential for these synergistic actions and multiple interactions with other neurotransmitter and neuropeptide systems underline the importance of OXT for the fine-tuned balance of emotionality, stress coping and complex social interactions, which shape our personality and mental health. The most promising indications from studies to date include the role of OXT in social behaviour, stress-attenuation and interactions with the serotonergic system. However, the studies to date are not conclusive with regard to the role of OXT in the aetiology and potential treatment of MDD. For example, we are only beginning to understand detailed molecular mechanisms of OXT actions at the neuronal level. Thus studies such as those demonstrating the detailed neuronal intracellular signalling pathways involved in the anxiolytic and spatial memory-enhancing effects of OXT [143,144] are necessary in the context of MDD. These studies should include the assessment of central OXT administration in chronic stress paradigms, in drug-withdrawal paradigms, and in conjunction with antidepressant administration. Moreover, assessment of the role of OXT in additional endophenotypes of depression is sorely warranted, for examine cognitive dysfunction and sleep disturbances under control and stess-altered conditions. Of interest, is the development of the non-peptidergic OXT agonist, WAY-267464, which replicated the anxiolytic, but not antidepressant-like, properties of OXT. Further studies with this, or similar, compounds would also substantially improve the assessment of the OXT system in MDD as they could be applied peripherally. This would solve a major problem of the human studies to date, which have assessed intra-nasal effects of OXT without verifying the levels in the brain. Interestingly, at present there is greater knowledge from human studies revealing OXT to be candidate system in the aetiology and treatment of MDD; at least at the level of certain endophenotypes. Replication of these studies, and translation to preclinical models would give greater cause for optimism in the use of OXT as an antidepressant with a novel mechanism of action. Thus, whereas CRF and AVP with their described anxiogenic and depression-like effects could represent Scylla and Charybdis in behaviour regulation, OXT like Circe counteracts these effects and mediates anxiolysis, calmness, rewarding and the positive consequences of social support [13,107]. 
